# Left brain cortical activity modulates stress effects on social behavior

**DOI:** 10.1038/srep13342

**Published:** 2015-08-25

**Authors:** Eunee Lee, Jiso Hong, Young-Gyun Park, Sujin Chae, Yong Kim, Daesoo Kim

**Affiliations:** 1 Biological Sciences, Korea Advanced Institute of Science and Technology (KAIST), Daejeon, 305-701, Korea; 2 Graduate School of Medical Science and Engineering, Korea Advanced Institute of Science and Technology (KAIST), Daejeon, 305-701, Korea; 3 Institute for the BioCentury, Korea Advanced Institute of Science and Technology (KAIST), Daejeon 305-701, Korea; 4 Laboratory of Molecular and Cellular Neuroscience, Rockefeller University, New York, New York 10065, USA

## Abstract

When subjected to stress, some individuals develop maladaptive symptoms whereas others retain normal behavior. The medial prefrontal cortex (mPFC) is known to control these adaptive responses to stress. Here, we show that mPFC neurons in the left hemisphere control stress effects on social behavior. Mice made socially avoidant by the stress of chronic social defeats showed depressed neural activity in the left mPFC. Photoactivation of these neurons reversed social avoidance and restored social activity. Despite social defeats, resilient mice with normal sociability showed normal firing rates in the left mPFC; however, photoinhibition of these neurons induced social avoidance. The same photomodulation administered to the right mPFC caused no significant effects. These results explain how stressed individuals develop maladaptive behaviors through left cortical depression, as reported in mood and anxiety disorders.

Stress is a risk factor for several mood disorders, including major depressive disorder^1^,^2^,^3^ and anxiety disorders like post-traumatic stress disorder (PTSD)^4^,^5^,^6^. Individuals exhibit varying degrees of susceptibility towards developing deleterious behavioral symptoms of mood disorders^7^,^8^. In a similar vein, mice exposed to chronic stress can be subdivided into two subgroups: one that develops deleterious behaviors like anhedonia, social avoidance or helplessness, and another that does not exhibit such characteristics^9^,^10^,^11^,^12^,^13^. Recent studies have sought to identify the neural mechanism by which stress induces maladaptive symptoms.

Lateralization of the medial prefrontal cortex (mPFC) in stress has been studied as focused on the modulation of stress hormones^14^, which has been implicated in behavioral deficits induced by chronic stress^15^,^16^. When subjected to chronic stress, the left mPFC hemisphere undergoes structural loss reflecting volume shrinkage^17^,^18^,^19^, whereas the right mPFC hemisphere plays a dominant role in facilitating stress hormone responses through interactions with the hypothalamic-pituitary-adrenal gland (HPA) axis^20^,^21^,^22^. Indeed, lesion of the right mPFC in rats causes stress resistance owing to a decrease in stress hormone levels^23^.

Another line of evidence has recently suggested that the mPFC is also involved in stress resilience by interacting with regions associated with reward pathways^24^,^25^, such as the ventral tegmental area (VTA)^26^,^27^. Stress-induced behavioral symptoms, manifesting as vulnerability or resilience to stress, can be modulated by pharmacogenetic^28^ or optogenetic^29^,^30^ manipulation of the mPFC. The responsiveness of mPFC neuronal activity to conspecific potential aggressors in naïve animals is a predictor of these animals’ tendency to display behavioral changes to chronic stress^31^.

Thus, the mPFC may play multiple roles in controlling stress responses, from stress hormone modulation to emotional and behavioral changes. Although the right mPFC hemisphere is known to predominantly control stress hormones in stressed animals^23^, whether other mPFC functions are also lateralized remains unclear. To address this issue, we examined neural activity in the two mPFC hemispheres in mice after administering chronic social defeat stress and compared this activity between mice in two distinct behavioral groups: those showing normal resilience and those showing extreme social avoidance.

## Results

### Hemispheric differences in mPFC neuronal firing between susceptible and resilient mice

**Figure 1:**
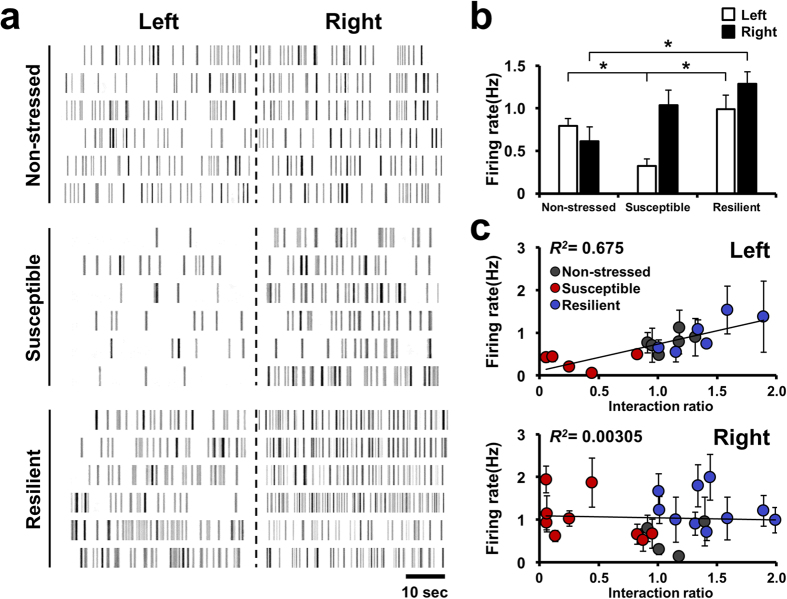
Distinct neuronal firing patterns in the left and right mPFC of stressed mice. (**a)** Representative unit firing of left and right mPFC excitatory neurons in non-stressed, susceptible, and resilient mice. (**b**) Comparison of mean firing rates of excitatory neurons between hemispheres. Total unit number/number of mice: non-stressed left, n = 54/6; non-stressed right, n = 21/5; susceptible left, n = 26/5; susceptible right, n = 76/9; resilient left, n = 25/6; resilient right, n = 85/9. Left mPFC mean firing rate in susceptible mice was different from that in non-stressed and resilient mice, whereas the right mPFC firing rate was increased in the resilient group compared with controls. Left mPFC (open bar): *F*_*2,14 *_= 7.355, *P *= 0.018, one-way ANOVA. Right mPFC (filled bar): *F*_*2,20 *_= 3.368*, P *= 0.055, one-way ANOVA. Non-stressed Left vs. Susceptible Left, t_9 _= 3.851, *P = 0.0039, t-test; Susceptible Left vs. Resilient Left, t_9 _= −3.356, *P = 0.00845, t-test; Non-stressed Left vs. Resilient Left, t_10 _= −1.039, *P *= 0.323, t-test; Non-stressed Right vs. Susceptible Right, t_12 _= −1.560, *P *= 0.145, t-test; Susceptible Right vs. Resilient Right, *P *= 0.158, Mann-Whitney Rank Sum test; Non-stressed Right vs. Resilient Right, t_12 _= −2.914, *P = 0.013, t-test. Data are presented as means ± s.e.m. **c,** Linear regression analysis showing that social preference indices were positively correlated with left mPFC neuronal activity (left), but not with right mPFC neuronal activity (right). Grey dots, non-stressed mice; red dots, susceptible mice; blue dots, resilient mice. Left mPC: *R*^*2 *^= 0.675, *F*_*1,15 *_= 31.123, *P *< 0.001, ANOVA. Right mPC: *R*^*2 *^= 0.00305, *F*_*1,21 *_= 0.642, *P *= 0.802, ANOVA. Data are presented as means ± s.e.m.

After undergoing chronic social defeat, stressed mice were divided into two subgroups according to their social approach behaviors to unfamiliar mice (Supplementary Table). One group exhibited social avoidance (interaction ratio < 1), defined as susceptible, whereas the other expressed normal social interaction behavior (interaction ratio > 1), defined as resilient^32^. Although social avoidance is known to be associated with mPFC activity^29^,^33^, functional differences between the two hemispheres in the manifestation of this behavior have not been investigated. To explore this question, we performed single-unit recording in mice subjected to 10 days of chronic social defeat stress (Fig. 1a, Supplementary Fig. S1). Neural firing rates of right mPFC neurons were higher in stress-exposed mice compared to those in naïve control mice (Supplementary Fig. S2), whereas there was no significant difference in right mPFC neuronal firing rates between susceptible and resilient subgroups of stress-exposed mice (Fig. 1b). These results are consistent with the idea that the right mPFC regulates stress hormone levels in response to stress^21^.

In contrast, activity in the left mPFC was significantly lower in susceptible mice than in resilient and non-stressed mice (Fig. 1b), suggesting that the change in left mPFC activity is related to the difference in sociability between the two subgroups. To confirm this idea, we compared the firing rates of each mPFC hemisphere in individual mice with their social interaction ratios. Interestingly, regression analysis revealed a linear correlation between firing rates in the left mPFC and social interaction ratio (Fig. 1c, upper panel), whereas firing rates in the right mPFC showed no such relationship with stress-induced changes in sociability (Fig. 1c, lower panel). Taken together, these results suggest that left mPFC neuronal activity is closely associated with the expression of stress-induced social avoidance behavior whereas firing rate of right mPFC is not involved in expression of stress-induced social avoidance behavior of mice.

### Photoactivation of the left mPFC abolishes social avoidance in susceptible mice

**Figure 2:**
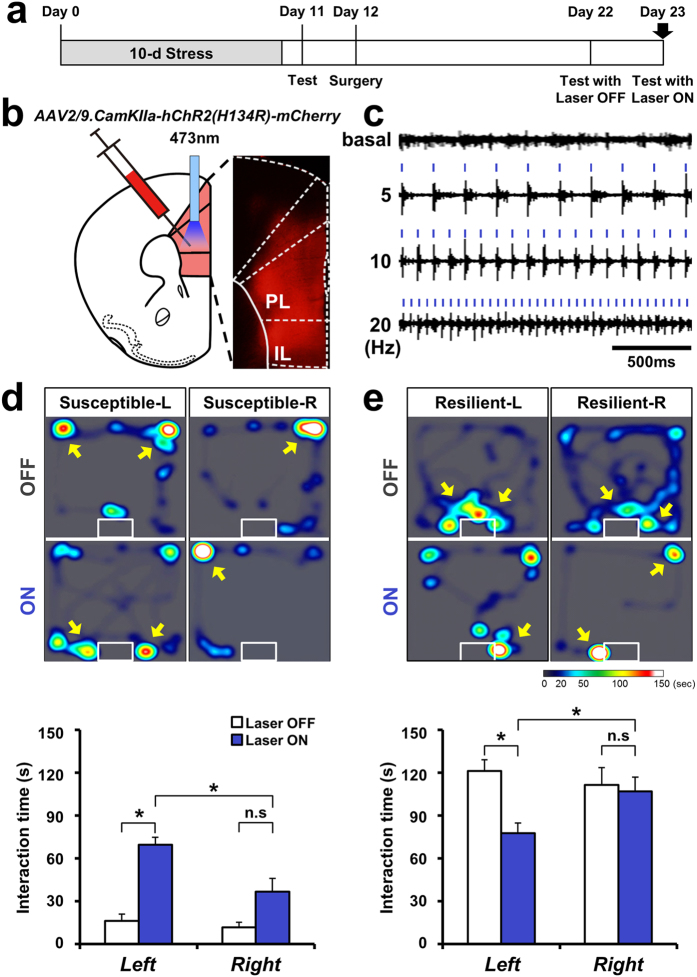
Recovery of sociability in susceptible mice by photostimulation of left mPFC neurons. (**a**) Timeline of optogenetic stimulation experiments. (**b**) Infection pattern of AAV2/9-CamKII-hChR2(H143R)-mCherry in the mPFC. A schematic of mPFC is drawn guided by Franklin and Paxinos mouse brain atlas. (**c**) Multiunit recordings following stimulation with 473-nm laser pulses of 5, 10, and 20 Hz. (**d)** Upper: Representative heat map indicating the location of susceptible mice without (OFF) or with (ON) stimulation in the presence of a social target. *Left panel:* Behavior of susceptible mice stimulated in the left mPFC (Susceptible-L). *Right panel:* Behavior of susceptible mice stimulated in the right mPFC (Susceptible-R). Lower: Comparison of social-interaction time in the presence of a social target among susceptible mice receiving left or right stimulation of the mPFC (Left, n = 5; Right, n = 5). Left stimulation restored social interaction in susceptible mice, whereas right stimulation had no significant effect. Left OFF vs. Left ON, t_4 _= −12.556, *P = 0.0002, paired t-test; Right OFF vs. Right ON, t_4 _= −2.533, *P *= 0.064, paired t-test; Left ON vs. Right ON, t_8 _= 3.129, *P = 0.014, t-test. Data are presented as means ± s.e.m. (**e**) Upper: Representative heat map indicating the location of resilient mice without (OFF) or with (ON) stimulation in the presence of a social target. *Left panel:* Behavior of resilient mice stimulated in the left mPFC (Resilient-L). *Right panel:* Behavior of resilient mice stimulated in the right mPFC (Resilient-R). Lower: Comparison of social-interaction time in the presence of a social target among resilient mice receiving left or right stimulation of the mPFC (Left, n = 6; Right, n = 5). Left stimulation decreased social interaction in the resilient phenotype, whereas right stimulation did not significantly change social interaction. Left OFF vs. Left ON, t_5 _= 4.384, *P = 0.007, paired t-test; Right OFF vs. Right ON, *P *= 0.625, Wilcoxon signed rank test; Left ON vs. Right ON, t_9 _= −2.479, *P = 0.035, t-test. Data are presented as means ± s.e.m.

To examine the roles of the right and left mPFC in controlling sociability after stress, we selected the most extreme cases of both susceptible (social-interaction time < 40 seconds) and resilient (social-interaction time > 100 seconds) animals. Optogenetic manipulation, successfully inducing or inhibit action potential with illuminating specific wavelength of laser^34^, was used to change neural activity of left and right mPFC of mice. The adeno-associated viral construct AAV2/9-CamKIIa-hChR2(H143R)-mCherry was unilaterally delivered to either the right or left prelimbic area of the mPFC in these selected mice. After 10 days, to allow adequate viral expression (Fig. 2a,b), pulses of laser stimuli (473 nm) at various frequencies (1–20 Hz) were administered at the site of injection. We found that a 10-Hz pulse stimulation was sufficient to induce robust neural firing without spike reduction due to adaptation^35^ (Fig. 2c).

Whereas all mice from the susceptible subgroup still exhibited high social avoidance behaviors after surgery, those that received a 10-Hz photoactivation of the left mPFC exhibited restoration of sociability to normal levels (Fig. 2d, Supplementary video S1). In contrast, photostimulation of susceptible animals injected in the right mPFC failed to induce any significant behavioral changes (Fig. 2d). In the resilient subgroup (Fig. 2e), photostimulation of the left mPFC decreased interaction time to a level similar to that of controls and stimulated susceptible mice (Fig. 2d, Supplementary Fig. S3), restoring social behavior in these mice to a normal range^32^ (Fig. 2e). In contrast, stimulation of the right mPFC did not change social interaction levels (Fig. 2e). The social behavior of non-stressed mice was not changed by photostimulation of the left mPFC (paired t-test) or right mPFC (Supplementary Fig. S3). These data collectively suggest that decreased left mPFC activity in susceptible mice (Fig. 1) is a critical factor for the expression of social avoidance, and that normal social behavior is rescued by an artificial increase in left mPFC activity.

### Photoinhibition of left mPFC activity leads to social avoidance in resilient mice

**Figure 3:**
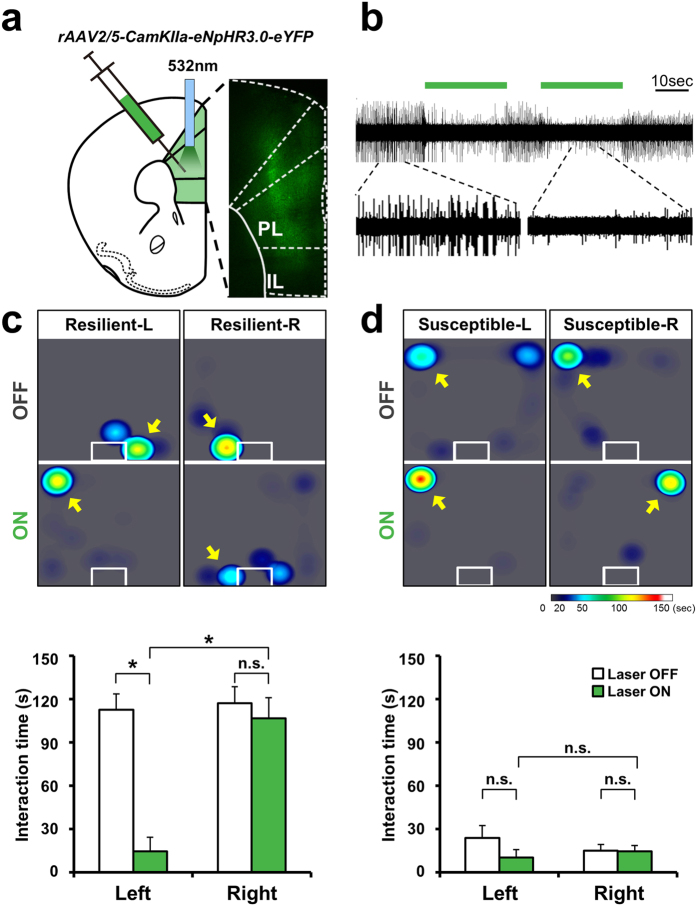
Induction of social avoidance in resilient mice by photoinhibition of left mPFC neurons. (**a**) Infection pattern of rAAV2/5-CamKII-eNpHR3.0-eYFP in the mPFC. (**b**) Neuronal firing was successfully inhibited with the eNpHR(3.0) system, using the same duration as was used in the behavioral paradigm. (**c**) Upper: Representative heat map indicating the location of resilient mice without (OFF) or with (ON) stimulation in the presence of a social target. *Left panel:* Behavior of resilient mice stimulated in the left mPFC (Resilient-L). *Right panel:* Behavior of resilient mice stimulated in the right mPFC (Resilient-R). Lower: Comparison of social-interaction time in the presence of a social target among resilient mice receiving left or right inhibition of the mPFC (Left, n = 5; Right, n = 7). Left inhibition induced social avoidance in resilient mice, whereas right stimulation did not significantly change social interaction. Left OFF vs. Left ON, t_4 _= 5.881, *P = 0.004, paired t-test; Right OFF vs. Right ON, t_6 _= 1.438, *P *= 0.201, paired t-test; Left ON vs. Right ON, t_10 _= −4.871, *P = 0.0007, t-test. Data are presented as means ± s.e.m. (**d**) Upper: Representative heat map indicating the location of susceptible mice without (OFF) or with (ON) stimulation in the presence of a social target. *Left panel:* Behavior of susceptible mice stimulated in the left mPFC (Susceptible-L). *Right panel:* Behavior of susceptible mice stimulated in the right mPFC (Susceptible-R). Lower: Comparison of social-interaction time in the presence of a social target among susceptible mice receiving left or right stimulation of the mPFC (Left, n = 4; Right, n = 6). Neither left nor right stimulation significantly changed social interaction. Left OFF vs. Left ON, t_4 _= 1.236, *P *= 0.284, paired t-test; Right OFF vs. Right ON, t_5 _= 0.138, *P *= 0.896, paired t-test; Left ON vs. Right ON, *P *= 0.329, Mann-Whitney U test. Data are presented as means ± s.e.m.

Next, we measured how social behavior in stressed mice changes in response to unilateral inhibition of mPFC activity. To this end, the left or right mPFC of stressed mice (Fig. 3a) that exhibited social behavior at the extremes of either resilience or susceptibility was unilaterally transfected with rAAV2/5-CamKIIa-eNpHR 3.0 (halorhodopsin 3.0)-eYFP virus. Targeted illumination with a 532-nm light pulse successfully inhibited action potentials in eNpHR3.0-expressing neurons (Fig. 3b). When such photoinhibition was applied to the left mPFC, resilient mice became socially avoidant, as evidenced by a dramatic reduction in the time spent within the interaction zone compared to that without stimulation (Fig. 3c, Supplementary video S2). The same procedure performed on susceptible animals elicited no changes in social avoidance (Fig. 3d). Photoinhibition of the right mPFC had no significant effect on sociability levels in either susceptible or resilient mice (Fig. 3c,d). Moreover, the social behavior of non-stressed mice was not changed by photoinhibition of either mPFC (Supplementary Fig. S4). These results imply that, despite the fact that resilient mice show normal levels of interaction even after stress, their expression of social avoidance is in fact being actively suppressed by left mPFC firing.

### The right mPFC has a role in the perception of social defeat stress

**Figure 4:**
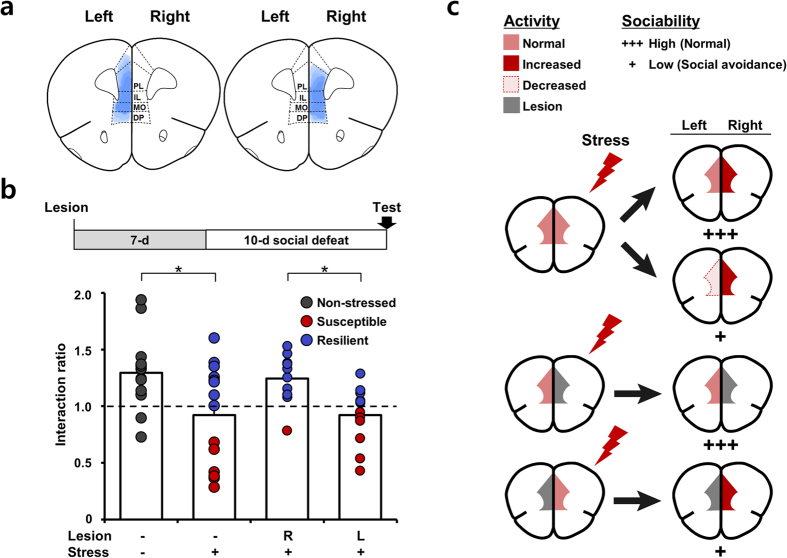
The effect of lesion in either of the two mPFC hemispheres on social defeat stress. (**a**) Regional extent of electrolytic lesion in a slice. (**b**) Timeline of social defeat stress after unilateral lesion of the mPFC. Interaction ratio (±social target) was measured in control and socially defeated mice without or with right (R) or left (L) mPFC lesion. Grey dots, non-stressed mice; red dots, susceptible mice; blue dots, resilient mice. Non-stressed controls, no lesion, no social defeat, n = 14; social defeat without lesion, n = 14; social defeat with right (R) lesion, n=10; social defeat with left (L) lesion, n = 12. Non-stressed vs. social defeat without lesion, t_26 _= 2.522, *P = 0.018, t-test; social defeat with right lesion vs. social defeat with left lesion, t_20 _= 3.166, *P = 0.005, t-test. Data are presented as means ± s.e.m. (**c**) Model of mPFC function in the regulation of social behavior in stressed mice and stress acquisition. Chronic social defeat stress induces an increase in the right mPFC, while the firing rate of the left mPFC changes differentially according to social behavior. In the susceptible group, low activity in the left mPFC results in expression of social avoidance. However, increased activity of the left mPFC in resilient mice reinstates social interactions to normal levels. Unilateral lesion of the mPFC before stress affects acquisition of stress. Right mPFC lesion prevents perception of stress, manifesting as stress resistance. On the other hand, lesion of the left mPFC blocks the expression of stress resilience, causing mice to show low sociability.

Given the apparent involvement of the left mPFC in arbitrating social interaction in response to stress (Figs 1, 2, 3), we naturally were curious about the role of neural activity in the right mPFC. Considering that stress hormone levels are a reliable indicator of an animal’s awareness of stress^15^ and the right mPFC hemisphere is closely associated with their regulation^22^,^23^,^36^, the higher levels of right mPFC neuronal activity in stressed mice compared to naïve mice (Supplementary Fig. 2) could reflect the acquisition of stress in an all-or-none manner. To verify this hypothesis, we performed the same assessments on mPFC-lesioned and normal mice. Mice in the lesioned group were administered unilateral electrolytic lesions to either mPFC hemisphere (Fig. 4a). As before, all groups were subsequently subjected to 10 days of social defeat conditioning, and their behavior was evaluated using an identical social-interaction procedure (Fig. 4b).

In line with the results described above, stressed mice without lesions segregated into susceptible and resilient subgroups (Fig. 4a,b), as previously reported^11^,^32^. More interestingly, mice with left mPFC lesions were mostly socially avoidant, shifting to the susceptible phenotype. In contrast, most subjects with damage to the right mPFC showed no social avoidance after stress (Fig. 4a,b), confirming that the right mPFC is critical for stress acquisition in a social defeat model, as was previously reported for a rat model of restraint stress^23^.

## Discussion

Previous studies have usually focused on the asymmetric contributions of the two hemispheres to the release of stress hormones^37^. Yet beyond this asymmetry, our data reveal that the two mPFC hemispheres also serve disparate functions at different stages in stress: right mPFC neurons appear to control the acquisition of stress during hazardous experiences, probably through the influence of stress hormone levels, as previously described; in contrast, activity in the left mPFC has a dominant role in translating this stress effect into social behavior.

In addition to stress acquisition and expression being segregated by hemisphere, these two modes of information are stored via differing mechanisms. Whereas right mPFC neurons show an all-or-none coding of information about stressful experiences, left mPFC neurons have a graded mechanism in which their firing rate reflects the degree of influence the stress has on behavior. Furthermore, we found that the information present in neural activity persisted ~5–10 days after the social defeat events. As such, information on neural activity in the two mPFC hemispheres makes it possible to predict whether a particular animal will be susceptible or resilient to a stressful experience.

This functional difference between mPFC hemispheres implies a mechanistic definition of stress resistance versus resilience. Photoinhibition of left mPFC neurons triggers the expression of social avoidance in resilient mice (Fig. 3). This means that despite these animals having ‘normal’ levels of sociability, they still may develop social avoidance; this behavior is simply suppressed by increased left mPFC activity. However, the right mPFC functions in a different manner. Photoinhibition of the right mPFC after social defeat conditioning fails to confer the resilient phenotype, although its lesioning beforehand does lead to stress resistance (Fig. 4). Thus, our model elegantly explains the mechanistic difference between stress ‘resistance’ and ‘resilience’ based on mPFC function^38^.

This raises the question, how are firing rates in left mPFC neurons correlated with behavioral phenotypes of stressed mice? On the one hand, it is likely that depressed left mPFC activity in susceptible mice reflects decreased interactions with resilient pathway regions, such as the VTA^11^,^24^,^39^. Consistent with this supposition, it is known that VTA neurons release dopamine to the mPFC^24^,^27^ and that dopamine depletion in the mPFC increases social avoidance^40^. An active supply of dopamine from the VTA^24^ in resilient animals may increase the excitability of mPFC pyramidal neurons^41^,^42^, thus enabling these animals to show normal levels of social activity^25^. Our results strongly suggest that the effects of this resilience mechanism are translated via neural activity in the left mPFC.

Not only inputs to mPFC, but outputs from mPFC have been reported to be involved in the regulation of stress responses^29^,^43^. Manipulation of mPFC activity using optogenetics has revealed the heterogeneous influence of mPFC outputs on behavior changes after stress: modulation of specific projections from the mPFC was shown to induce pro-resilient effects, whereas stimulation of other projections failed to restore stress behavior^30^,^43^,^44^. Our study indicates that the left mPFC is predominantly involved in these resilience-related projections.

Another potential explanation is that mPFC activity directly controls sociability. An imbalance between excitation/inhibition (E/I) in the mPFC is known to contribute to the development of sociability deficits^45^. Extreme depression of the left mPFC in susceptible mice would provoke an E/I imbalance between the two hemispheres of the mPFC and induce behavioral deficits. Social isolation, which results in structural changes in mPFC neurons^46^, leads to a reduction in social activity^47^. This effect of social isolation on behavior can also be explained by a depression in left mPFC activity.

On the basis of our results, we propose a bi-stable model to explain how the development of deficits in social behavior is influenced by cortical lateralization^48^,^49^ (Fig. 4c). Under stress conditions, the right mPFC plays a dominant role in acquisition of stress, whereas the left mPFC determines the expression of chronic-stress–influenced (social) behavior. The left mPFC is able to appropriately regulate such behavior independent of the activity of the right mPFC, and confers resilience. Thus, future studies investigating the mPFC in relation to mood and anxiety disorders should employ experimental designs capable of assessing the two distinct cortical hemisphere systems separately. The identification of molecules that selectively control left or right mPFC activity could be a viable strategy for developing therapeutics for mood disorders.

## Methods and Materials

### Animals and behavioral tests

C57B/6J male mice (13–15 weeks old) were used in all experiments. Mice were maintained with free access to food and water under a 12:12-hour light:dark cycle. Chronic social stress was administered as reported previously^32^. All animal care and experimental procedures were performed in accordance with protocols approved by the directives of the Animal Care and Use Committee of Korea Advanced Institute of Science and Technology (approval number KA2012-04).

Social-interaction tests^39^ were performed inside a dark room in a box (42 × 42 × 42 cm) with white walls and an iron mesh cage (6 × 6 × 10 cm) at one wall to house the social target. The social-interaction test was recorded as a digital file, and the time spent in the interaction zone was analyzed using Ethovision XT (Noldus). The social preference index (interaction ratio) was obtained by calculating the ratio of time spent in the interaction zone in the presence of the target to that in the target’s absence. Stressed mice were categorized as susceptible or resilient based on their time inside the target-interaction zone, with those spending less than 40 seconds defined as susceptible and those spending more than 90 seconds as resilient.

### Electrolytic lesion

Mice were anesthetized with Avertin (2, 2, 2-tribromoethanol; Sigma) and fixed on a stereotaxic device (Kopf Instruments). An electrolytic lesion was created at a site in the left or right hemisphere of the mPFC (distance from bregma, ±2.10 mm; medio-lateral axis, ±0.3 mm; depth from the pia mater, 2.50 mm) by administering a 1-mA current for 15 seconds using a monopolar stainless steel electrode (A–M System 564410) and a stimulus isolator (A365; World Precision Instruments). After 1 week of recovery, mice were subjected to a 10-day chronic social defeat stress paradigm. On the last day of the stress schedule, a social-interaction test was administered to the stressed mice as described above. The region of the electrolytic lesion was visualized by harvesting and fixing the mouse brain in 4% formaldehyde, then cryosectioning (30 μm thickness) and staining with cresyl violet. After comparing the damaged region with Franklin and Paxinos mouse brain atlas^50^, any mice showing effects of the electrolytic lesion in the secondary motor cortex (M2), ventral orbital cortex (VO), or corpus callosum were excluded.

### Optogenetic stimulation of mPFC neurons

Two AAV constructs, one expressing an mCherry fusion of the hChR2 (H134R) variant [AAV2/9-CamKIIa-hChR2(H134R)-mCherry] and the second expressing a YFP fusion of eNpHR3.0 [rAAV2/5-CamKIIa-eNpHR3.0-eYFP], both under control of the neuron-specific CaMKIIa promoter, were obtained from Penn Vector Core (USA). AAV2/9-CamKIIa-hChR2(H134R)-mCherry and rAAV2/5-CamKIIa-eNpHR3.0-eYFP, with titers of 4.09 × 10^1 ^particles/ml and 4 × 10^12 ^particles/ml, respectively, were used as previously described, with minor modifications^51^,^52^.

AAV2/9-CamKII-hChR2(H134R)-mCherry or rAAV2/5-CamKII-eNpHR3.0-eYFP was injected into the left or right mPFC area (distance from the bregma, +2.20 mm; lateral, ±0.30 mm; ventral, 1.5 mm), depending on the specific experiment described in the text. The optical fiber (200 μm diameter, 2 mm length) was inserted at the injection site in the form of a ferrule (D280-2350; Doric, Canada) and fixed to the skull with three surface screws and dental cement. This procedure was performed within 48 hours of the end of the chronic social defeat stress paradigm. On the tenth day after surgery, the social-interaction test was administered with optic fiber, without receiving laser stimulation.

On the next day, the mouse, with the optical fiber attached to a fiber-optic connector (FC/PC connector), was habituated to the home cage for 10 minutes and then tested for social interaction while receiving optical stimulation (10 Hz, 5 ms). Mice injected with hChR2 were stimulated at 473 nm (~1 mW) in conjunction with a stimulus isolator (S48, Grass), and eNpHR3.0 mice were stimulated at 532 nm (~1.3 mW) using a diode-pumped, solid-state laser (Shanghai Laser). Before every behavioral test with optical stimulation, the light intensity was measured using an optical power meter (Thorlabs, Germany).

The locations of virus injection and optical stimulation were confirmed by postmortem histology, guided by Franklin and Paxinos mouse brain atlas^50^. Mice exhibiting signs of seizure or abnormal motor behavior (muscle twitching, head nodding, or jumping), suggesting improper targeting of the optical fiber, were excluded from analysis.

### Electrophysiological recordings *in vivo*

From 5 to 10 days after the social-interaction test, the animals were fixed on a stereotaxic device under urethane anesthesia (1.5 g/kg). Their body temperatures were monitored and maintained using a temperature controller (Homothermic Blanket System; Harvard Apparatus). Quartz-coated tetrodes (0.5–2 MΩ; Thomas Recording) or 16-channel silicon probe (2–3 MΩ, Neuronexus) were placed at left and right hemispheres of the mPFC (distance from bregma, +2.10 to +1.70 mm; medio-lateral axis, ±0.3 mm; dorsal-ventral axis, 1.3 to 2.5 mm). The recording sites were localized by briefly dipping the tips of the tetrodes in a fluorescent dye solution (DiI, 50 mg/ml; Sigma) before tissue penetration, and the electrode tracks in brain slices were visualized under a confocal microscope using a rhodamine filter, as previously described^53^. Signals were amplified using an AC programmable-gain main amplifier (Thomas Recording), sampled at 30 kHz (DT3010; Neuralynx), and filtered at either 300–6000 Hz (for measurement of multiunit activity) or 1.52–50 Hz (for measurement of local field potentials). Spike sorting was performed using SpikeSort3D (Neuralynx), and units were classified as excitatory or inhibitory neurons by waveform analysis, as previously described^54^.

To confirm that the optogenetic stimulation was working, we performed multiunit recording with tetrodes 10 days after virus injection. Exposure of the tetrode to 473-nm laser (0–10 mW) confirmed that the laser did not induce a photoelectric current on the electrode. The multiunit recording procedure was the same as that described above for tetrode recordings. Additionally, an optical fiber was separately inserted as close as possible to the tetrode (within 0.5 mm). For NpHR(3.0), laser stimulation was given from the surface of brain. Recordings were obtained for 20 minutes without stimulation, and then optical stimulation and recording were performed simultaneously for another 20 minutes. No signal was detected from a non-injected mouse tested using the same protocol, even when a 10-Hz stimulation was given, confirming that the data were not false signals or electric signals from the stimulator.

## Additional Information

**How to cite this article**: Lee, E. *et al.* Left brain cortical activity modulates stress effects on social behavior. *Sci. Rep.* **5**, 13342; doi: 10.1038/srep13342 (2015).

## Supplementary Material

Supplementary Information

Supplementary video S1

Supplementary video S2
