# Supplementary material for: Left brain cortical activity modulates stress effects on social behavior
Source: Sci Rep. 2015 Aug 25;5:13342. doi: 10.1038/srep13342 (PMC4548443; doi:10.1038/srep13342)

Left brain cortical activity modulates stress effects on social behavior

Eunee Lee[1,2†], Jiso Hong[1†], Young-Gyun Park[1], Sujin Chae[1], Yong Kim[3], and Daesoo Kim[1*]

[1]Biological Sciences, Korea Advanced Institute of Science and Technology (KAIST), Daejeon, 305-701, Korea. [2]Graduate School of Medical Science and Engineering, Korea Advanced Institute of Science and Technology (KAIST), Daejeon, 305-701, Korea. [3]Laboratory of Molecular and Cellular Neuroscience, Rockefeller University, New York, New York 10065, USA.

†These two authors contributed equally to this work

*Correspondence to:

Daesoo Kim (daesoo@kaist.ac.kr)

Associate Professor

Behavioral Genetics Lab

Department of Biological Sciences

Korea Advanced Institute of Science & Technology (KAIST)

291 Daehak-ro, Yuseong-gu

Daejeon 305-701

Office: 82-42-350-2639

Supplementary Table. Behavioral characteristics of mice used in behavioral experiments.

	Ratio	Interaction Time (sec)	Locomotion (cm)
**Control**	1.521 ± 0.120	77.231 ± 4.005	2055.060 ± 73.385
**Resilient**	1.365 ± 0.061	111.175 ± 2.053	1622.662 ± 49.629
**Susceptible**	0.493 ± 0.049	48.176 ± 5.842	1703.658 ± 79.129

Supplementary figures and legends

Supplementary figure S1. Neuronal classification and location of recordings in single unit recording. a, Classification of mPFC neuron types by using waveform analysis; the height of first and second peak (a and b) and duration between first valley to second peak (c) (inset) are used to identify excitatory (Ex) or inhibitory neurons (In) left and right mPFC. **b,** Electrode location after post hoc analysis marked by DiI. Numbers are the distance from the bregma on anterial-posterial axis.

Supplementary figure S2. Increased firing rate of the right mPFC in stressed mice. The firing rate of neurons in the right mPFC is increased after being subjected to chronic social defeat stress regardless of their behavioral phenotype. Total unit number/recorded mice: control left, n=54/6; control right, n=21/5; stressed left, n=51/11; stressed right, n=149/16). Non-stressed right vs. Stressed right, t_{21}=-2.313, **P*=0.0309, t-test; Stressed left vs. Stressed right, **P*=0.014, Mann-Whitney U test. Data are presented as means ± s.e.m.

Supplementary figure S3. Non-stressed mice do not show difference in social behavior after 10Hz photostimulation. Left, n=9; Right, n=10. Left Laser ON vs. Right Laser On, t_{17}=-0.831, *P*=0.418, t-test; Left Laser OFF vs. Left Laser ON, t_8=0.353, *P*=0.733, paired t-test; Right Laser OFF vs. Right Laser ON, t_9=0.474, *P*=0.647, paired t-test. Data are presented as means ± s.e.m.

Supplementary figure S4. Non-stressed mice did not show difference in social behavior with photoinhibition. Left, n=6; Right, n=7. Left Laser ON vs. Right Laser On, t_{11}=1.761, *P*=0.106, t-test; Left Laser OFF vs. Left Laser ON, t_5=-0.460, *P*=0.665, paired t-test; Right Laser OFF vs. Right Laser ON, t_6=0.809, *P*=0.450, paired t-test. Data are presented as means ± s.e.m.

Supplementary videos

Supplementary video S1. Optogenetic stimulation of left mPFC in susceptible mice. A representative video clip of social interaction test of a susceptible mouse expressing AAV2/9-CamKIIa-hChR2(H143R)-mCherry at the left mPFC hemisphere. The mouse showed depressed sociability during light off, but with 473nm laser stimulation (light on), became active in social interaction.

Supplementary video S2. Optogenetic inhibition of left mPFC in resilient mice. A representative video clip of social interaction test of a resilient mouse expressing rAAV2/5-CamKIIa-eNpHR3.0-eYFP at the left mPFC hemisphere. The mouse showed normal social activity during light off, but with 532nm laser stimulation (light on), became socially avoidant.

Supplementary Figure 1.

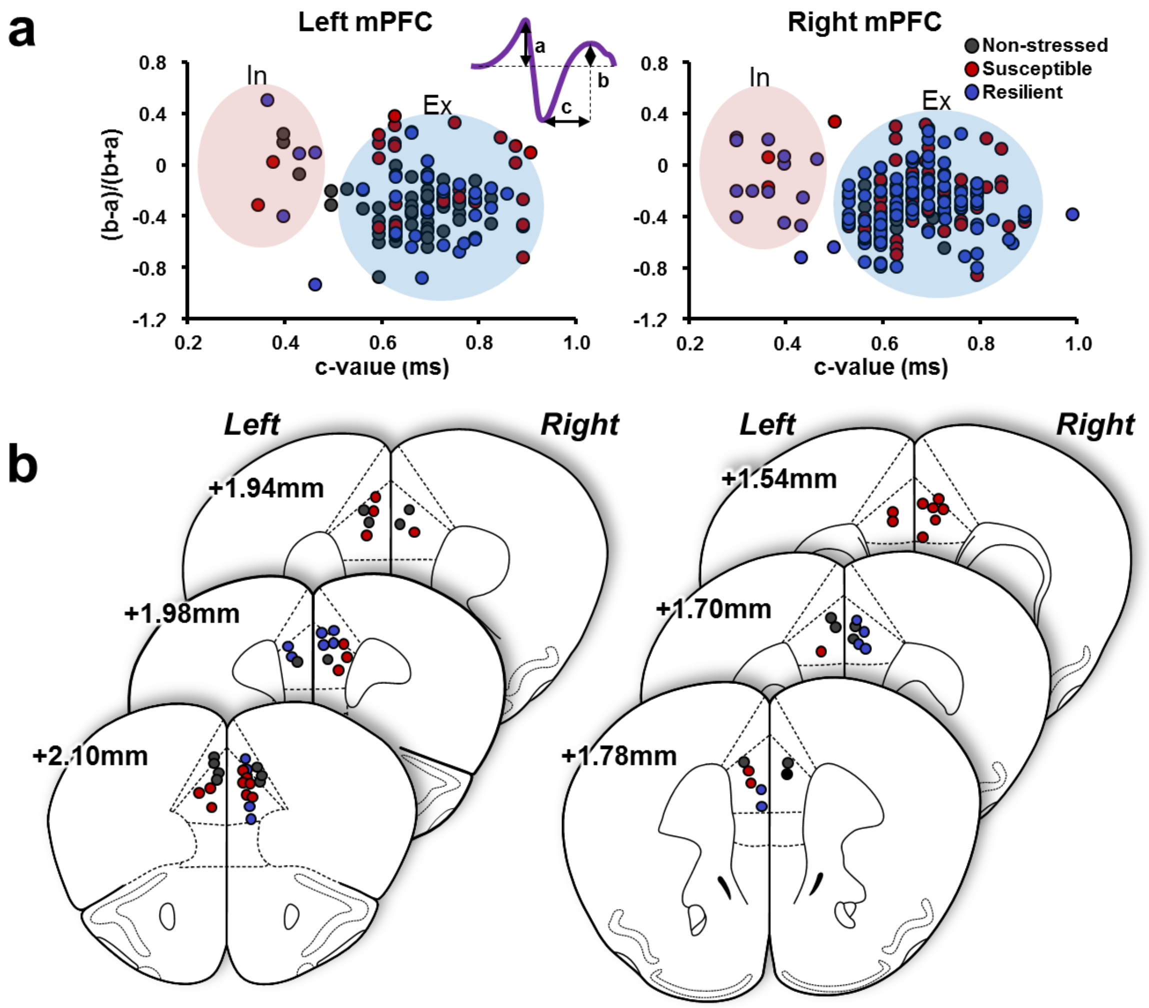

Supplementary Figure 2.

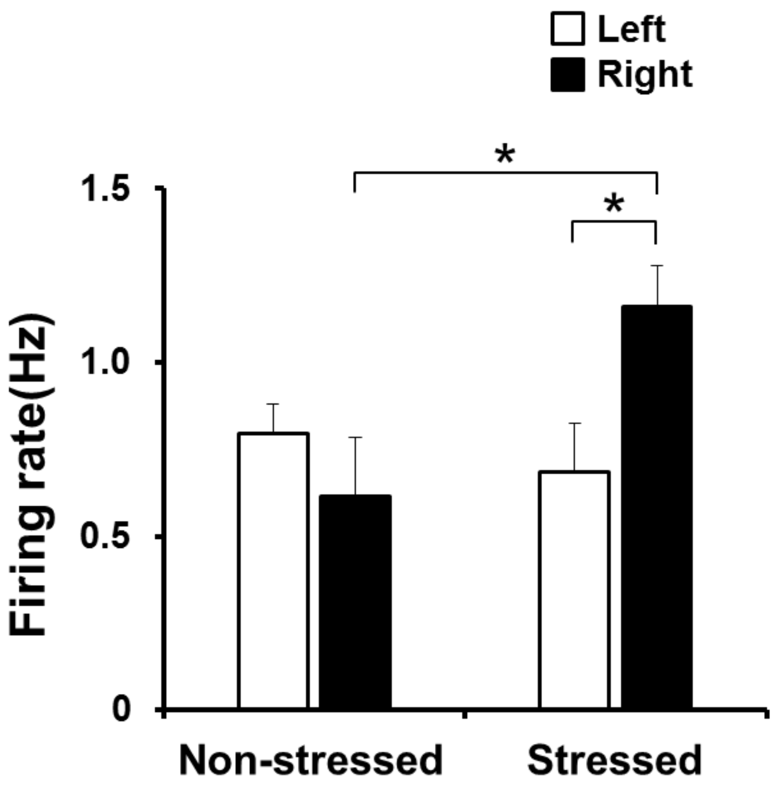

Supplementary Figure 3.

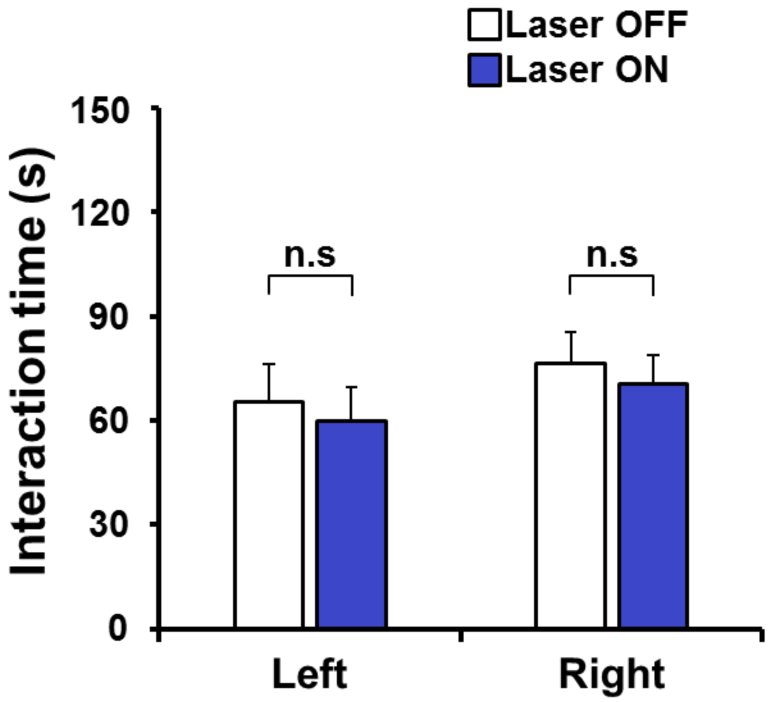

Supplementary Figure 4.

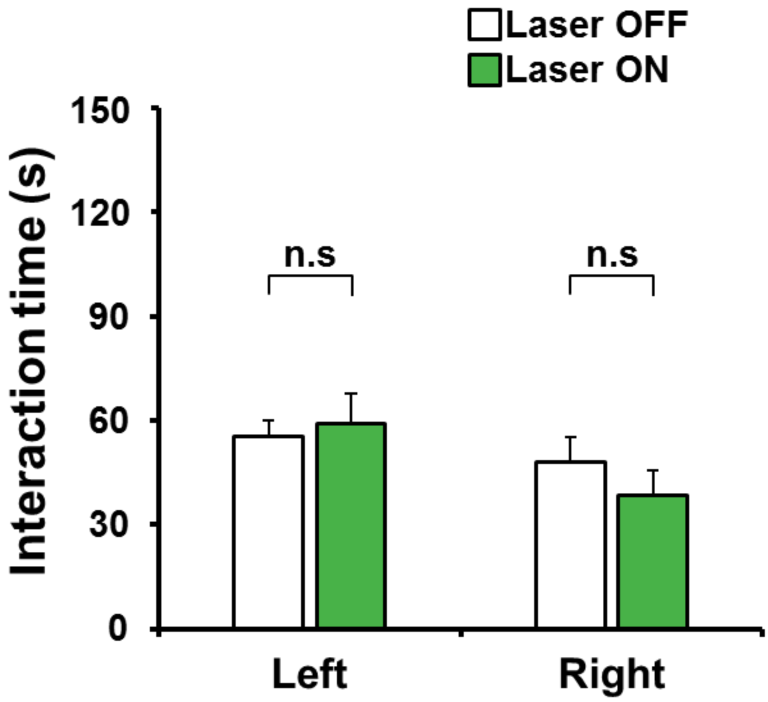

Supplement: Supplementary Information [file srep13342-s1.pdf]
